# Experiences of ecosystem changes on food services of mopane woodland communities in Vhembe, South Africa

**DOI:** 10.1007/s10661-024-13115-x

**Published:** 2024-09-20

**Authors:** Andisa A. Mufungizi, Walter Musakwa, Nelson Chanza

**Affiliations:** Department of Geography, Environmental Management & Energy Studies, University of Johannesburg, Johannesburg, Gauteng South Africa

**Keywords:** Indigenous food plant, Shifting ecosystem, Mopane woodlands, Climate variability, Adaptation

## Abstract

Mopane woodlands have been shifting. While it is important to understand the spatial patterns that characterise this phenomenon, it is even more important to understand the impacts of shifting Mopane woodlands on rural communities that rely on them. This study sought to establish the impacts of shifting mopane woodlands on the production of indigenous plant food in Ward 12 of Musina local municipality in the Vhembe District municipality in the Limpopo province of South Africa. To accomplish this, the study utilised a hybrid inductive approach involving thematic-based questionnaire interviews and an exploratory view to gain insight into the narratives of focus group participants. Results revealed that seven (7) out of eleven (11) indigenous plant foods are becoming extinct, thereby limiting food sources of indigenous and local people who used to rely on them. The spatial pattern of the plant foods that are still available has now changed as they no longer grow within the reach of local communities. The community members are struggling to adapt to these changes. From these observations, we recommend that local and regional levels’ policies related to natural resource management should consider the unique challenges faced by communities experiencing disruptive ecosystem changes and provide the necessary support for sustainable adaptation.

## Introduction

Over 10% of the world’s population lives in unacceptable conditions of poverty. These populations are mostly located in rural areas of low-income, developing countries(Khan et al., 2020). This, therefore, makes the study of natural ecosystems very urgent in these parts of the world because these populations still rely on food obtained directly from these sources. Moreover, studies have shown that the attainment of many sustainable development goals (SDG) is directly and indirectly linked to the quality, quantity or availability of ecosystem services which influence the direct or indirect benefits that humans derive from ecosystems. In particular, ecosystem services can help achieve SDGs related to the environment (Wood et al., 2018). For example, Qiu et al. (2022) found that provisioning services and regulating services have positive direct influences on human well-being. In respect of SDGs 1 and 2 which stand for no poverty and zero hunger respectively, ecosystem services provide resources and livelihoods for millions of people, especially for rural and indigenous communities. They are also viewed as nature’s contribution to the well-being of humans and their quality of life (Fisher et al., 2009; Hernández-Blanco et al., 2022). Therefore, ensuring the biodiversity of ecosystems and their health and productivity would help address the issue of poverty and hunger. In terms of SDG 3 which stands for good health and well-being, ecosystem services contribute to human health through clean air and water, medicinal resources, and disease regulation.

There is growing scientific evidence of changes in natural ecosystems (Drinkwater et al., 2010; Fang et al., 2022; Nelson et al., 2006; Prakash, 2021). These changes manifest in different forms, including the decline in ecosystem services (Fang et al., 2022) and biodiversity loss (Habibullah et al., 2022; Schmeller et al., 2020). Several factors constitute the driving forces for biodiversity loss and ecosystem services decline. These include, but are not limited to, habitat destruction and fragmentation (Adla et al., 2022; Li et al., 2022; Pal et al., 2021), climate change (Forzieri et al., 2022; Shivanna, 2022; Weiskopf et al., 2020), pollution (Kumar et al., 2021), overexploitation (Gacheno & Amare, 2021; Khorrami & Malekmohammadi, 2021), invasive species (Rai & Singh, 2020), and land use change (Fang et al., 2022; Hasan, et al., 2020; Zhao et al., 2020).

The shifting of ecosystems is also one of the ways in which ecosystems have responded to climate change and anthropogenic activities and has been observed in both terrestrial and marine ecosystems. Furthermore, scientists in disciplines such as ecology, environmental studies, evolutionary biology and geobotany have reported evidence of this phenomenon. Concepts such as “regime shift” (Demirel et al., 2023) and “range shift” (Charitonidou et al., 2021) dominate the shifting ecosystem literature. The term shifting ecosystem is not commonly used in literature, but studies revolving around regime shifts and range shifts in ecosystems point to the reality of shifting ecosystems. Such ecosystems can be described in terms of their composition, manifestation, structure and function (Defeo et al., 2021; Ma et al., 2019). In terms of composition, a shifting ecosystem can be characterised by biodiversity loss and movement in the spatial occurrences of plant and animal species. These spatial changes in locations can lead to the loss of biodiversity in certain ecosystems while increasing biodiversity in other ecosystems. Shifting ecosystems also undergo changes in their fundamental structure (Parmesan et al., 2022). This could be a result of invasive species or other factors such as climate change, human activities, and geological forces. The restructuring of shifting ecosystems may also manifest in terms of vegetation loss to invading plant species. Therefore, studies are currently investigating how habitat colonisation is affecting ecosystem functions and processes, leading to the restructuring of ecosystems. This is very important, because habitat colonisation has a direct impact on the re-organisation of biotic components of ecosystems, be they plants or animals. For an ecosystem to be considered as shifting, it must have reached a tipping point or threshold. This is a point where an ecosystem moves away from one stable state to another (Whyte, 2020).

Little research has studied the social impacts of shifting ecosystems, especially in the context of rural communities. The structure and functioning of rural communities is in most cases, an outcome of the natural resources available to them through ecosystem services (Li et al., 2019). For this reason, it is expected that any negative changes to ecological systems supporting these communities would have a direct impact on the lived experiences of people (Rimal et al., 2019).

The supply of good quality healthy food from the natural ecosystem is therefore a factor that influences the well-being of people to a large extent (Mao et al., 2019). In the case of rural communities, the supply of indigenous food directly from nature is of paramount importance because it is not only healthy, but it also forms part of the identity of the people (Trott, 2023). An understanding of the impacts of shifting ecosystems on the supply of indigenous food from natural ecosystems such as the mopane woodland is, therefore, important. Such an understanding would help improve ecosystem management practices in rural areas, chart new pathways to livelihoods and most importantly, it would help develop effective climate change adaptation strategies amongst other benefits.

Several studies have been undertaken on mopane woodlands and on the Vhembe district. These have endeavoured to investigate possible barriers to the adoption of climate change adaptation strategies such as Climate-Smart Irrigation Technologies amongst smallholder farmers (Serote et al., 2023). Some have attempted to document indigenous knowledge about the use of medicinal and food plants in the mopane woodlands (Urso et al., 2016). However, none of these has sought to investigate the impacts of shifting ecosystems on the production of plant foods or even to characterise the nature of shifting mopane woodlands. They have also not sought to identify how mopane woodland-dependent communities perceive the impacts of shifting ecosystems on their indigenous foods, and how best these communities could adapt to these changes.

The objective of this study was therefore to determine the social impacts of the shifting mopane woodland. This was done by establishing the impacts of these shifts on the ecosystem supply of indigenous plant food in five villages of Ward 12 of Musina local municipality in the Vhembe district of the Limpopo province. Assessing the impacts of shifting ecosystems on plant food is considered a means of determining the social impacts of shifting mopane woodland because of its role in ensuring human well-being. The Intergovernmental Panel on Climate Change (IPCC) defines human well-being as “a state of existence that fulfils various human needs, including material living conditions and quality of life, as well as the ability to pursue one’s goals, to thrive, and feel satisfied with one’s life” (Matthews, 2018). Human well-being has many facets to which food contributes either directly or indirectly. They include basic material needs for a good life, health, good social relations, security, and freedom of choice and action. In terms of basic material needs for a good life, food security can be directly linked to economic productivity by which people access the materials they need. The proper nutrition and/or balanced diet can also prevent diet-related diseases such as obesity and diabetes. Food also provides nutrients that are necessary for the growth, development and maintaining of bodily functions. In terms of good social relations, food has cultural significance. Continuous availability of indigenous food is of paramount importance to a community that identifies such food as an avenue through which their culture finds expression. Food, therefore, can strengthen the bond of a community and foster a sense of belonging. Finally, food security contributes to the overall sense of security of an individual and a community. It brings stability and provides a basis for a community to thrive.

The mopane woodlands are the most dominant vegetation type in Southern Africa and in the northern parts of South Africa in particular(Kashe et al., 2023). The mopane woodlands have often been referred to as social woodlands because of their diverse and unique flora with actual and potential environmental, social, and economic benefits (Maquia et al., 2019; Nikodemus et al., 2023). These woodlands provide ecosystem services and livelihood benefits that support the lives of many people, especially in rural areas (Bara et al., 2022). The mopane woodlands are used for fuel, poles, medicine, mopane worms, stink bugs, termites, edible locusts, thatching, and sweeping grasses, which are harvested to meet household needs for food, energy, and income generation (Lavhelani, 2021).

## Materials and methods

### Study area

**Fig. 1:**
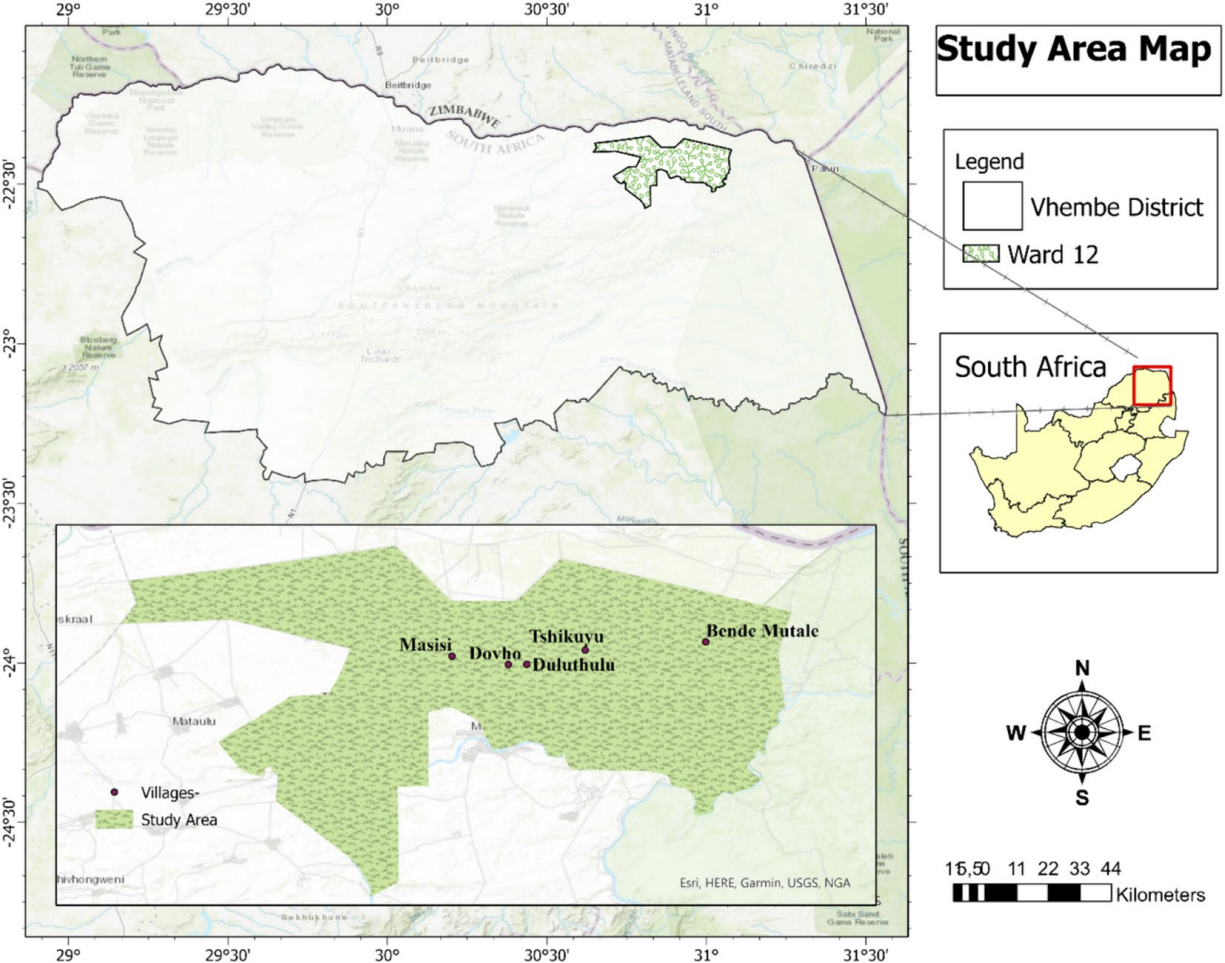
Map of Ward 12

The Bende Mutale, Masisi, Tshikuyu, Duluthulu and Dovho villages are in the northern part of South Africa, in Ward 12 of the Musina local municipality in the Vhembe district of the Limpopo province (Fig. 1). The five villages are situated along the R525 road from Makhado leading to the Pafuri gate of the Kruger National Park. The Vhembe district has 89% of its population classified as rural (Rusere et al., 2019). It has a subtropical climate with temperatures ranging from a minimum of 10 °C in winter to a maximum of 40 °C in summer. Furthermore, food security and hunger persist in the district, with females’ income being less than males. This perpetuates gender income inequality, with 70% of people living under the food poverty line (Vhembe District Municipality, 2020). This background makes the Vhembe District an interesting subject in investigating the social impacts of shifting ecosystems. According to Mpandeli and Maponya (2013), Vhembe is affected by long dry spells which grow into severe drought conditions.

The five villages were chosen because they are located in the heart of the Angolan mopane woodlands in the district. According to Mucina and Rutherford (2006) and White (1983) in Makhado et al. (2012), the two types of woodlands in Southern Africa consist of the Zambezian and the Angolan mopane. Only the Angolan mopane woodlands are found in South Africa (northern part). Some Angolan woodlands cover a portion of the Vhembe district, where the 5 villages are located, while other Angolan mopane woodlands are located within the Kruger National Park. Hence, the five villages were of special interest for this study because communities living in these villages are well positioned to tell the story of how the mopane woodland has been shifting and what the social impacts of this shift have been.

### Data collection

This study determined the social impacts of the shifting mopane woodland on the ecosystem supply of indigenous plant food in five villages of Ward 12 of Musina local municipality in the Vhembe district of the Limpopo province. To achieve this goal required the identification of the indigenous food sources. Most importantly, it was imperative to determine what changed in terms of the quantities (availability) and quality in the indigenous foods obtained from the mopane woodland. Therefore, a mixed method was adopted utilising a questionnaire-based survey and qualitative focus group discussions (FGDs). The FGDs were conducted with 13 community members at the Bende Mutale village. Though the discussions took place in one village, the participants came from all five villages, and the Bende Mutale village was simply the meeting place. All of them were adults with ages ranging between 30 and 65 years. This selection was based on the assumption that the participants have been living in the study area for a long period and have therefore witnessed the changes in the food that is produced from the mopane woodlands. Thus, the discussions focused on the types of food obtained from the mopane woodlands and the changes that have been happening over the years in the quality as well as the quantity of the food.

A questionnaire-based survey was conducted in 2022 with 137 community members across the five villages. This was preceded by a pilot survey carried out in August 2022 with 50 community members. The pilot study helped determine the eagerness of community members to participate, and how important the topic of indigenous food was to them. We used the results of the pilot study to restructure the questionnaire and to ensure that important aspects of the study were covered. These included the identification of existing indigenous foods in the study area as well as those indigenous foods that have become extinct, and the perception of community members about the changes in the quality and quantity of these foods. Also, the pilot study made it easier to discern aspects which needed further probing when conducting the actual interviews and the FGDs.

The survey participants were identified randomly, but their age group determined whether they were to be considered for the interview. Participant’s personal information, such as names and physical addresses, was not requested. However, demographic information such as gender, employment status, education level, and age were collected. These were necessary as they helped glean how the various age groups perceived the impacts of the shifting mopane woodlands on the food produced from the mopane woodlands.

Local research assistants were engaged across the five villages. For the FGD in the Bende Mutale village, an audio recording was made, and this discussion was coordinated to ensure that every FGD participant had a chance to talk. This was done so that the most talkative participants would not dominate the conversation. Participants were allowed to speak in both English and their local language. When participants expressed themselves in their local language, the research assistants assisted with translating into English. The interview questionnaires were written in English, and the audio was later transcribed into English where necessary. The local research assistants were first trained on the research instruments so that they would get familiar with the study and be able to grasp the research protocol.

FGD participants were asked to identify the types of food they had been obtaining from the mopane woodland. This was followed by the question about what might have changed in the availability of these foods and their quality over the past 30 years. By means of questionnaires, respondents were asked to select the types of foods which they obtained from the mopane woodland. These foods were initially identified through the pilot study. They were also asked to indicate what has changed in terms of the taste, quality and quantity in the foods they obtain from the woodland. Based on the findings of the pilot study, interview respondents were also asked to select statements that best described the changes that have occurred to the foods produced by the mopane woodland. They were also asked to identify the foods which are no longer available because of the changes that have occurred in the mopane woodland.

In addition to the data gathered through questionnaires and the FGDs, climate data, i.e. daily maximum temperature, daily minimum temperature, and precipitation (mm), was obtained from the South African Weather Services. Data from the Mara meteorological station was considered because it is the closest station to the study area. This data was necessary to allow correlation between the perceptions of community members about changes in the indigenous plant foods and the change in climate of the study area from the year 1982 to 2022.

Some limitations were encountered when conducting this study, such as the language barrier between the community members (participants) and the researchers. This challenge was tackled by employing research assistants from amongst communities who then served as mediators between research participants and the researchers. It is possible that there might have been some level of bias in the way research participants responded to research questions but one can only trust that the participants responded to the best of their knowledge and ability.

### Data analysis

An inductive approach to narrative analysis was employed together with an exploratory view to gain insight into the stories of the focus group participants. An inductive thematic approach was also employed to analyse the data collected. This helped identify the patterns in the responses of participants. In this study, narrative was considered as any meaningful utterance (spoken or written) or topical stories about the impacts of shifting mopane woodland on the quality and the quantity of indigenous food supplied by the mopane woodland. The view given by Franzosi (1998:517) was embraced that describes narratives as texts that are “packed with sociological information, and a great deal of our empirical evidence is in narrative form”. In this study, an interpretive approach to narrative analysis was followed, as the focus was more on the content and not necessarily the structure of narratives. ATLAS.ti 23 software was used to do the thematic analysis of open-ended questions of the survey and the response of FGD participants. ATLAS.ti 23 offers an Artificial Intelligence (AI) coding feature that makes thematic analysis efficient. Use of ATLAS.ti generated codes which we reviewed and modified, based on the knowledge of the data and the interactions in the field during data collection. The review of the codes was also done in consideration of the research objectives. Once the codes had been reviewed, these were analysed with the objective of identifying patterns that would lead to themes. Prior to the research being done, an ethical clearance certificate was obtained from the Ethics and Plagiarism Committee of the Faculty of Engineering and the Built Environment at the University of Johannesburg under Ethical Clearance Number UJ_FEBE_FEPC_00537.

## Results and discussion

### Availability of indigenous food

A total of 11 different indigenous fruits were identified from the mopane woodland (Table 1). A participatory analysis with the study participants further described in the fruits in terms of quantity, quality, and availability. Quantity and availability were considered as one category according to the narratives provided by the participants that revealed the correlations between them.

**Table 1:** Indigenous foods obtained from the mopane woodland

No	Local name	English name	Scientific name
1	Mbuyu	Baobab fruit	*Adansonia digitata* L. (Malvaceae)
2	Mafula	Marula fruit	*Sclerocarya birrea*
3	Mahuhuma	Shakama plum	*Hexalobus monopetalus*
4	Mazwilu	Wild medlar	*Vangueria infausta*
5	Thaladzi	Milkwood or transvaal red milkwood	*Mimusops zeyheri*
6	Tsuma	African ebony	*Diospyros mespiliformis*
7	Tshikili	Forest natal-mahogany	*Trichilia emetica*
8	Nthu	Blackberry	*Rubus fruticosus*
9	Thololwe	-	-
10	Bungavhuselo	-	-
11	Nie	-	-

First, it was found that the quantity of fruits produced from the mopane woodlands has decreased, especially since the year 2000. FGD participants traced the significant changes in the availability of these indigenous fruits to the year 2000. Though there were already changes prior to the year 2000, the most significant of these were noticed after this year. The year 2000 was very significant to the community because of the floods that occurred (Munyai et al., 2021). According to them, changes in the frequency of fruit production, the harvest quantity and subsequently the quality were more pronounced in the years that followed the 2000 floods. For example, the FGD participants outlined that “some of the fruits are no longer growing regularly. They could grow one year and not grow the following year. Sometimes they only grow after two years when there is rain”.

Secondly, it was found that the quality of the fruits produced from the mopane woodland has also changed gradually. According to the FGD participants, foods produced from the mopane woodland no longer taste as they used to. The taste of foods seems to be the most significant change in the quality of foods produced from the mopane woodland. This was followed by the physical quality of foods, that is, the appearance of the fruits. Some of the fruits are drier than they used to be in the past. The change in the quality of fruits was also translated into the size of the food products from the mopane woodland (Greven et al., 2009). This could be because of droughts in the study area.

When asked about the statements that best describe the changes in the fruits obtained from the mopane woodland, 95,6% of the interview respondents indicated that “fruits that used to grow close to our houses are now growing far from our community”. This is crucial to the spatial pattern of the shifting mopane woodland ecosystem. This pattern implies that the community members are experiencing diminished access to fruits. About 74% of the participants corroborated that “the soil is no longer rich for agriculture due to erosion resulting from shortage of trees”. The declining agricultural potential of the soil in the community is likely to make adaptation to changes in the fruits from the mopane woodland difficult.

Of the 11 identified indigenous fruits, 7 were becoming extinct in the area (Table 2). This was confirmed by 62.5% of the interview respondents who indicated that “Some food products are no longer available”. This observation is in line with what was mentioned by the FGD participants when they identified seven (7) of their indigenous fruits as “no longer available”. These fruits are Mazwilu (Wild Medlar), Thaladzi (Milkwood), Tsuma (African ebony), Tshikili (Forest Natal-mahogany), Nthu (Blackberry), Thololwe, and Bungamutselo. This could have a negative impact on how the local community expresses their culture because these fruits are a significant aspect of their way of life. Indigenous foods are a strong link between cultural identity and the environment (Akinola et al., 2020). Therefore, losing some of these plant foods could result into the loss of cultural identity. This is so because of the beliefs and rituals that are often associated with indigenous fruits.

**Table 2:** Indigenous fruits that are no longer available in Ward 12

No	Local name	English name	Scientific name
1	Mazwilu	Wild medlar	*Vangueria infausta*
2	Thaladzi	Milkwood or transvaal red milkwood	*Mimusops zeyheri*
3	Tsuma	African ebony	*Diospyros mespiliformis*
4	Tshikili	Forest natal-mahogany	*Trichilia emetica*
5	Nthu	Blackberry	*Rubus fruticosus*
6	Thololwe	-	-
7	Bungamutselo	-	-

The loss of the indigenous fruits can also translate into nutritional decline with the potential to negatively impact the health of local residents. These natural fruits are believed to have superior nutritional value. The loss of these fruits is also an indication of biodiversity harm within the mopane woodland. This could have severe impacts on the functioning of the ecosystem, but exploring the possible disruptive impacts of these losses was beyond the scope of this study. Also, the loss of these indigenous fruits is having a negative economic impact on the local community members who are struggling to adapt to these changes because they now need to find new ways to supplement the dietary and nutritional loss. These new ways include buying food from retail stores. The challenge with this new reality is the rate of unemployment in these villages. Most of the residents rely on government grants for survival, but it is not enough. Though the soil is not the most fertile for agricultural purposes, community members are resorting to subsistence farming. However, this is hampered by poor infertile soils. Moreover, though not very pronounced in this study, the loss of traditional knowledge and techniques associated with the processing of these indigenous fruits is another possible impact on the community.

### Spatial patterns in indigenous fruits from the mopane woodlands and drivers of shifting mopane woodland

The FGD revealed that there have been changes in the spatial patterns of the indigenous fruits obtained from the mopane woodland. It was found that the indigenous plant foods that are still available in the study area no longer grow near to where residents reside. This pattern could be explained by changes in the soil properties that favour the growth of these indigenous foods and changes in the occurrences of climatic variables such as minimum and maximum temperature and precipitation. Though it was beyond the scope of this study to investigate specific factors behind the changes in the spatial patterns that now characterise the spatial occurrences of the indigenous fruit trees, the narratives given by FGD participants suggest that the shifting of the mopane woodland is evident.

Further, upon analysing the data obtained through the perception surveys, it was revealed that there are five (5) factors influencing the spatial shifting of the mopane woodland ecosystem and which contribute to the decline and extinction of indigenous plant foods. These are deforestation, droughts, growth conditions of trees, limited rainfall, and lack of soil nutrients. The community members concur that deforestation and lack of soil nutrients are the major factors contributing to the shifting of the mopane woodland.

### Correlating climate change to the community’s perceptions about changes in indigenous plant foods

**Fig. 2:**
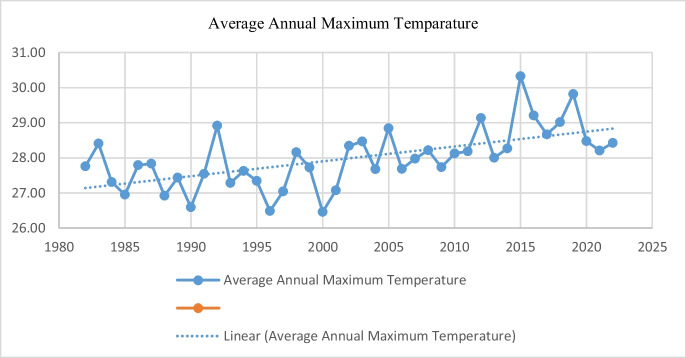
Average annual maximum temperature

**Fig. 3:**
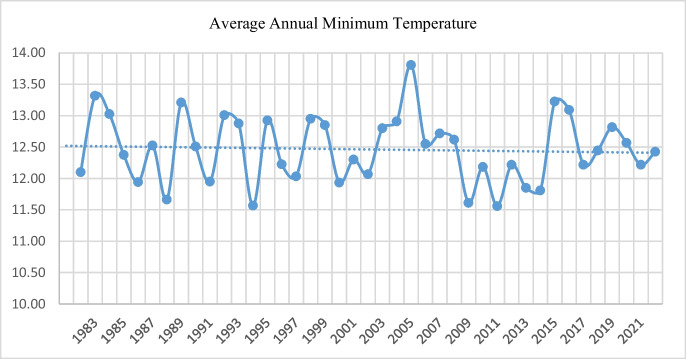
Average annual minimum temperature

Figures 2 and 3 outline the variations in average annual maximum and minimum temperatures for the study area. It appears that trends in average annual maximum temperature have increased accompanied by a significant variability. A similar trend has also been observed in the average annual minimum temperature whereby the minimum annual temperatures are decreasing but with high variability. Therefore, it can be said that perceived climate change in the study area is not necessarily as a result of sharp increases in annual maximum temperatures or sharp and gradual decreases in annual minimum temperatures. Rather, the climate change being experienced in the area is more of a result of high variability in annual temperatures.

**Fig. 4:**
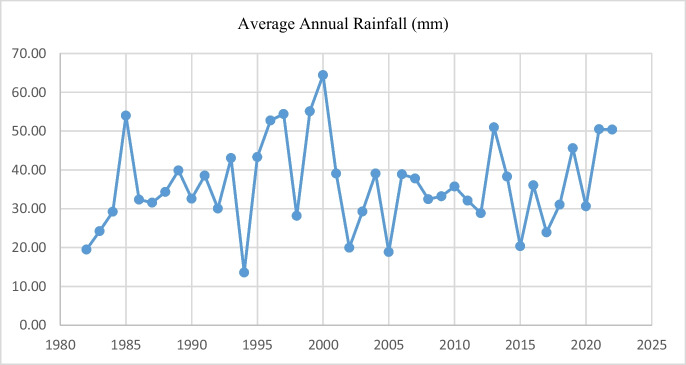
Average annual rainfall (mm)

When considering the changes in average annual precipitations, Fig. 4 shows that there has been a continuous decrease in annual rainfall, especially from the year 2000 as compared to previous years. This supports the perceptions of community members who related the scarcity of certain plant foods to the flood that took place in the year 2000. The community members went on to indicate that rainfall patterns also changed in subsequent years and Fig. 4 is evidence of this.

### Adaptation strategies to changes in indigenous fruits

A uniform response of survey participants was observed when they were asked the following question: “How are you adapting to the changes in the food you used to get from the woodland ecosystem?”. Their common response was that “it is not easy to adapt”. Three (3) adaptation themes emerged from the analyses done in ATLAS.ti 23. These are adjusted diet, relocation, and farming (Table 3).

**Table 3:** Adaptation themes related to adapting to changes in indigenous fruits

No	Themes	Description
1	Adjusted diet	The consumption of foods bought from local spaza shops
2	Relocation	Some of the residents now relocated to urban areas, especially in towns that are closely located to the five villages
3	Farming	Residents who choose to stay in the community practice subsistence farming. Some of them cultivate vegetables for domestic consumption and rear cattle which they sell to support their families

The functioning of these local communities has been altered around food consumption in response to the changes that have taken place in the fruits obtained from the mopane woodland. First, community members are now learning to depend on food sold in spaza shops. These are food items which the community perceives as being unhealthy because they are not obtained directly from their natural ecosystem. Secondly, it was found that there is an ongoing trend of relocation, whereby some community members are forced to leave their respective villages to resettle in nearby towns because of the inability to cope with the changes in the fruits previously obtained from the mopane woodland. Thirdly, it was found that some of the community members decided to stay in their respective villages and practised subsistence farming. This has been a daunting adaptation response because of the drastic changes that have happened to the climate of the area over the past 30 years. The scarcity of water and the low rainfall that now characterises the area makes it difficult to practice farming (Shikwambana et al., 2021).

Further, the mopane woodlands play a role in carbon sequestration and maintaining local climate conditions. The observed changes can disrupt these functions, potentially exacerbating climate change effects and reducing the resilience of the local environment. Mopane woodlands are also home to diverse plant and animal species, many of which are adapted to specific microhabitats. The changes reported herein have probably led to habitat loss and a decrease in species diversity, thus affecting ecological interactions and leading to the possible extinction of indigenous species. These changes could lead to the spread of invasive species, which can could alter the ecological balance, leading to further biodiversity loss.

Furthermore, these realities call for urgent action in ensuring a more sustainable and long-term liveability of the area. These realities also call for ecosystem management and conservation initiatives to safeguard the ecological integrity of mopane woodlands and other climate-sensitive ecosystems. These initiatives could be in the form of policies promoting the reforestation of the woodlands with the objective of preserving their biodiversity. The reforestation should therefore focus on replanting species of trees which have become extinct. However, such an effort would be met with the challenge of climate change characterised by increasing temperatures and declining precipitations. Therefore, saving the biodiversity of the mopane woodlands would require a solution to the decreasing precipitation first.

Moreover, there is a need to conduct a participatory vulnerability mapping. Through this, the community can be involved in identifying the degree to which various sections of the community are vulnerable to declining ecosystem services and biodiversity loss caused by the shifting mopane woodland and its drivers. Already the socio-ecological context of Ward 12 outlines a growing prevalence of poverty and food insecurity associated with the decline of ecosystem services, especially food plants, and the biodiversity loss. Therefore, the vulnerability mapping exercise would be a good starting point for developing adaptation strategies and policy interventions with lasting effects. Similar to identifying community vulnerability, the population should also be involved in profiling biodiversity losses. The findings of this study have pointed to the possibility of biodiversity loss in other climate-sensitive ecosystems in the area. Ward 12 and the Vhembe district at large have a history of being rich in biodiversity of fauna and flora. This study shows that it is time that the decline in ecosystem services, and the biodiversity loss should be quantified with the help of the communities living in these ecosystems. This would form the basis for better ecosystem management and conservation practices.

In addition, there is a need to promote interdisciplinary research characterised by collaboration between local communities, the local government as well as other government agencies active in the area. It should include non-governmental organisations, and research institutions to address the complex socio-ecological challenges emanating from shifting mopane woodlands. This would help develop adaptation strategies that can lead to more specific and targeted adaptation efforts. Also, seeing that many indigenous plant fruits no longer grow in the study area, it will be good to preserve the cultural heritage of these communities. This heritage is often directly connected to the indigenous foods (source). This could involve the documentation of traditional practices, rituals, and knowledge related to these foods.

## Conclusion

This study determined the social impacts of the shifting mopane woodland on the ecosystem supply of indigenous plant foods in five villages of Ward 12 of Musina local municipality in the Vhembe district of the Limpopo province of South Africa. This was done by employing an exploratory inductive approach to narrative analysis with a view to gain insight into the stories of focus group participants. An inductive thematic analysis was also conducted on data collected through questionnaires and focus group discussions. Eleven (11) indigenous plant foods were identified as being very significant to the local communities. Seven of these plant foods were reported as becoming extinct by community members. It was also found out that the spatial pattern of the growth of indigenous fruits that are still available in the study area has also shifted. These fruits are becoming less abundant and are now found far away from the community. The local people are not passive observers of these changes, but their adaptation mechanisms include adjusted diet, relocation, and farming. However, the communities are failing to effectively adapt to these changes. The decline in the quality and availability of plant foods also calls for ecosystem management and conservation initiatives to safeguard the ecological integrity of mopane woodlands and other climate-sensitive ecosystems in the area. These initiatives could be in the form of policies promoting the reforestation of the woodlands with the objective of preserving the biodiversity of the woodlands. Furthermore, it is recommended that interdisciplinary research should be undertaken to better understand the diverse socio-ecological problems emanating from the shifting mopane woodlands. These types of studies would also help encourage communities whose livelihoods are predominantly reliant on climate-sensitive woodland ecosystems to develop appropriate adaptation interventions to climatic disruptions.

## Data Availability

No datasets were generated or analysed during the current study.
